# Reactive Oxygen Species: A Promising Therapeutic Target for *SDHx*-Mutated Pheochromocytoma and Paraganglioma

**DOI:** 10.3390/cancers13153769

**Published:** 2021-07-27

**Authors:** Katerina Hadrava Vanova, Chunzhang Yang, Leah Meuter, Jiri Neuzil, Karel Pacak

**Affiliations:** 1 Section of Medical Neuroendocrinology, *Eunice Kennedy Shriver* National Institute of Child Health and Human Development, National Institutes of Health, Bethesda, MD 20892, USA; katerina.hadravavanova@nih.gov (K.H.V.); lmeuter@stanford.edu (L.M.); 2 Institute of Biotechnology, Czech Academy of Sciences, BIOCEV, Vestec, 252 50 Prague West, Czech Republic; jiri.neuzil@ibt.cas.cz or; 3 Neuro-Oncology Branch, Center for Cancer Research, National Cancer Institute, National Institutes of Health, Bethesda, MD 20892, USA; chungzhang.yang@nih.gov; 4 School of Pharmacy and Medical Science, Griffith University, Southport, QLD 4222, Australia

**Keywords:** reactive oxygen species, succinate dehydrogenase, metastatic pheochromocytoma, paraganglioma

## Abstract

**Simple Summary:**

Pheochromocytoma and paraganglioma are rare neuroendocrine tumors that arise from chromaffin cells of the adrenal medulla or their neural crest progenitors located outside the adrenal gland, respectively. About 10–15% of patients develop metastatic disease for whom treatment options and availability are extremely limited. The risk of developing metastatic disease is increased for patients with mutations in succinate dehydrogenase subunit B, which leads to metabolic reprogramming and redox imbalance. From this perspective, we focus on redox imbalance caused by this mutation and explore potential opportunities to therapeutically target reactive oxygen species production in these rare tumors.

**Abstract:**

Pheochromocytoma (PHEO) and paraganglioma (PGL) are rare neuroendocrine tumors derived from neural crest cells. Germline variants in approximately 20 PHEO/PGL susceptibility genes are found in about 40% of patients, half of which are found in the genes that encode succinate dehydrogenase (SDH). Patients with SDH subunit B (*SDHB*)-mutated PHEO/PGL exhibit a higher likelihood of developing metastatic disease, which can be partially explained by the metabolic cell reprogramming and redox imbalance caused by the mutation. Reactive oxygen species (ROS) are highly reactive molecules involved in a multitude of important signaling pathways. A moderate level of ROS production can help regulate cellular physiology; however, an excessive level of oxidative stress can lead to tumorigenic processes including stimulation of growth factor-dependent pathways and the induction of genetic instability. Tumor cells effectively exploit antioxidant enzymes in order to protect themselves against harmful intracellular ROS accumulation, which highlights the essential balance between ROS production and scavenging. Exploiting ROS accumulation can be used as a possible therapeutic strategy in ROS-scavenging tumor cells. Here, we focus on the role of ROS production in PHEO and PGL, predominantly in *SDHB*-mutated cases. We discuss potential strategies and approaches to anticancer therapies by enhancing ROS production in these difficult-to-treat tumors.

## 1. Introduction

**Figure 1:**
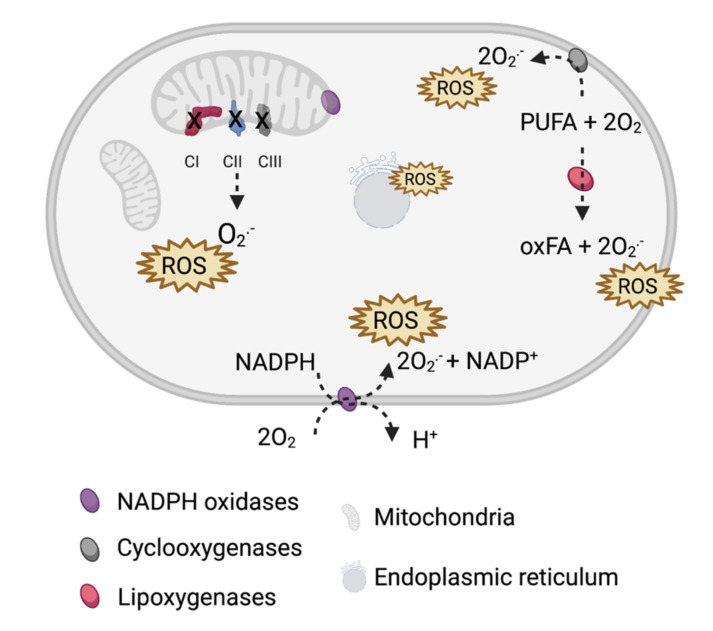
Main sources of reactive oxygen species (ROS) in cells. ROS are primarily generated by electron leaks from mitochondrial complexes I–III (CI–CIII) and ROS-producing enzymes: NADPH oxidases, cyclooxygenases, and lipoxygenases that catalyze the oxygenation of polyunsaturated fatty acids (PUFA). Additionally, protein oxidation on the endoplasmic reticulum (ER) and ER-related stress add to the ROS pool.

Reactive oxygen species (ROS) are oxygen-containing molecules with high reactivity including hydroxyl and superoxide radicals as well as superoxide non-radical molecules such as hydrogen peroxide. In eukaryotic cells, ROS are typically produced aerobically in mitochondria. Alternatively, ROS can be generated in peroxisomes via ß-oxidation of fatty acids and in the endoplasmic reticulum via protein oxidation (Figure 1). 

Growing evidence suggests that ROS (usually considered a metabolic waste) could serve as regulators of essential signaling and metabolic pathways. Continuous ROS production helps maintain control of cell proliferation and differentiation. Antioxidant enzymes closely regulate ROS production based on a scavenging system that is crucial for protecting eukaryotic cells from oxidative damage and other pathological processes including tumorigenesis [1,2,3]. Although cancer cells thrive on slightly higher levels of ROS compared to normal cells [3,4,5,6], cancer cells are more sensitive to external stimuli, which leads to significant redox adaptation and cell death induction [5,7]. Currently, anticancer therapy agents used to induce levels of ROS have been introduced in pancreatic, breast, colon, rectal, bladder, lung, and prostate cancers as well as melanoma, glioblastoma, and lymphoma (reviewed in [8]). From this perspective, we will focus on ROS production in pheochromocytoma (PHEO) and paraganglioma (PGL) and explore potential opportunities to therapeutically target ROS production in these rare tumors.

## 2. ROS in PHEO and PGL

PHEO and PGL are rare neuroendocrine neoplasms derived from chromaffin cells [9]. These tumors have a strong genetic disposition including variants in many Krebs cycle enzymes such as succinate dehydrogenase (SDH), fumarate hydratase, and malate dehydrogenase, which affect energy homeostasis and hypoxia signaling in cells [9,10]. Such defects in the Krebs cycle can result in accumulation of succinate and fumarate, which contribute to cancer development [11]. *SDH* mutations are found in approximately 17–28% of PHEO/PGL cases [12,13,14,15,16,17,18]. In particular, SDH subunit B (S*DHB*)-mutated PHEO/PGL is associated with a more aggressive tumor behavior and higher rates of metastatic disease when compared to all other PHEO/PGL cases (reviewed in [19]). These mutations alter the function of mitochondrial complex II and result in succinate accumulation, thus directly modifying oxidative phosphorylation and increasing ROS production in the *SDHx*-mutated tumor [20]. Currently, treatments for metastatic *SDHB*-related PHEO/PGL are palliative with very limited clinical benefit. There is an urgent need for more transformative therapies in order to improve disease outcomes in patients with *SDHB*-related PHEO/PGL. Recently, Moog et al. suggested angiogenesis, pseudohypoxia, epigenetics, metabolic reprogramming, and redox imbalance as therapeutic target options for the treatment of SDH-deficient PGLs [21]. 

**Figure 2:**
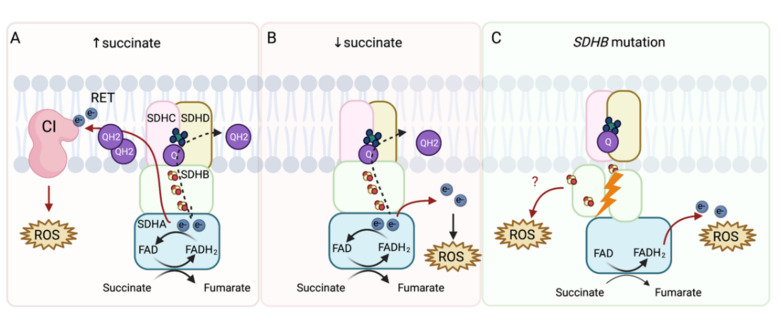
ROS production from SDH under physiological and pathophysiological conditions. The SDHA subunit covalently binds flavin adenine dinucleotide (FAD), which removes electrons from succinate to form fumarate. SDHB then transfers electrons via three iron–sulfur (FeS) clusters to the ubiquinone molecule (Q) located in the ubiquinone-binding site, formed by the SDHB, SDHC, and SDHD subunit. Ubiquinone is further reduced to ubiquinol, fueling CIII and CIV (dashed arrow). (**A**) At high succinate concentrations (≥5 mM), succinate-derived electrons reduce the ubiquinone pool and electrons are forced backward to CI in a process called reverse electron transfer (RET), which leads to indirect ROS production. (**B**) At physiological succinate concentrations, ROS can be produced directly. FAD is reduced to FADH2, which then reacts with oxygen within an unoccupied binding site to form ROS. (**C**) In cases of *SDHB* mutation, incorrect assembly of SDH can result in ROS production directly from reactions between oxygen and FADH2 or exposed FeS clusters.

Mitochondrial complex II significantly contributes to ROS production directly and indirectly via reverse electron transfer in CI [20,22,23,24,25], suggesting that this pathway could be used as a potential target in tumors with compromised SDH (Figure 2). Indeed, pharmacologic inhibition of SDHB in rat PHEO cells (PC12 cells) led to increased ROS production [26], which is consistent with findings from transgenic mouse cell line (NIH3T3 cells) with *SDHC* mutations [27] and Chinese hamster models of fibroblasts expressing truncated SDHC protein [28]. These findings resulted in the discovery of mitochondrial complex II as a target for cancer therapy [29,30]. Additionally, *SDHD*-mutated cDNA introduced into Chinese hamster fibroblasts resulted in increases of superoxide production with subsequent increases in mutation frequency and rate. This demonstrates the role of SDH-produced ROS in genomic instability within mammalian cells [31]. Furthermore, Guzy et al. found that SDHB inhibition (Hep3B human hepatoma cells, A549 human alveolar epithelium-derived tumor cells, and 143B human osteosarcoma cells) led to the accumulation of ROS [32], which was later confirmed by *SDHB* silencing in rat PHEO cells (PC12 cells) [33], mouse PHEO cells (MPC cells) [34,35], immortalized mouse chromaffin cells [36], and human hPheo1 cells [34,35]. Moreover, an increase in mitochondrial ROS production was observed in our model of the *SDHB* knock-out in human PHEO cell line (hPheo1, unpublished data). 

## 3. Targeting of ROS Production in PHEO and PGL

Recently, several studies have uncovered ways to suppress PHEO/PGL cell growth by ROS manipulation. Inhibition of pyruvate dehydrogenase kinase, either alone or in combination with an inhibitor of mitochondrial complex I (CI) metformin, resulted in oxidative metabolism promotion and intracellular ROS accumulation in immortalized head and neck PGL cell cultures from two patients carrying the *SDHC* and *SDHD* mutations [37]. This treatment promoted cell cycle arrest and apoptosis, which are the processes previously associated with ROS accumulation [37]. The increase in oxidative stress was confirmed in *SDHB*-silenced PHEO mouse models (MPC cells) and resulted in an increased demand for antioxidative defense [35]. In this study, the authors targeted glutathione (GSH) de novo synthesis with a nuclear factor erythroid-2 related factor 2 (NRF2) inhibitor. The targeted disruption of ROS homeostasis with the NRF2 inhibitor, brusatol, suppressed the growth of metastatic lesions from *SDHB*-silenced mouse PHEO cells (MPC) in vivo and prolonged overall survival of mice [35]. In addition to the significant ROS production, *SDHB*-silenced tumor cell lines (MPC and human pheochromocytoma precursor cells; hPheo1) accumulate iron, whereby treatment with ascorbic acid disrupts redox hemostasis, leading to ROS overload. This ultimately results in cell death in vitro and delays tumor growth in vivo [34]. Recently, dysregulation of iron homeostasis and ascorbate-induced accumulation of ROS resulting in cell death has been observed in *SDHB*-mutated immortalized chromaffin cells [36].

Interestingly, Pang et al. found that *SDHB*-mutated PHEO/PGL tumor tissue and *SDHB^KD^* PHEO cell culture (mouse metastatic tumor tissue; MTT) had increased mitochondrial complex I activity, which catalyzes the first step of the electron transport chain and oxidizes NADH to NAD^+^ [38]. NAD^+^ activates poly (ADP-ribose) polymerase (PARP), which stimulates DNA repair mechanisms and protects against DNA damage-associated cell death [39]. Inhibition and depletion of PARP activity leads to DNA damage and ROS induction [40]. Indeed, in *SDHB*-silenced PHEO models (MTT cells), PARP inhibition with olaparib in combination with a chemotherapeutic agent effectively reduced tumor proliferation, metastasis, and aggressive phenotypes in vivo [38]. These studies suggest that both increasing ROS accumulation to override the tolerated ROS pool and/or targeting the protective antioxidant mechanisms can suppress PHEO/PGL progression.

## 4. Future Directions

**Figure 3:**
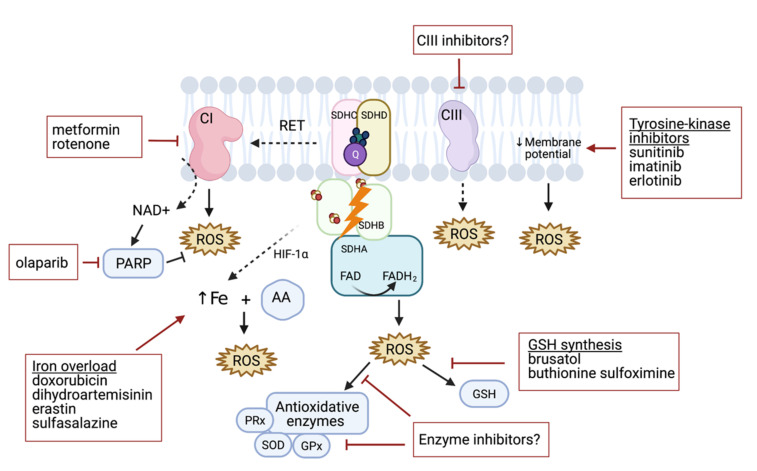
Targeting different mechanisms to induce and enhance ROS production in PHEO/PGL. ROS are generated by mitochondrial complex CI–CIII dysfunction, which may be further supported by CI, CII, and CIII inhibitors. Similarly, decreasing the membrane potential can lead to ROS accumulation. CII-related ROS production can be enhanced by increasing the iron pool and consequently increasing ROS production under ascorbate (AA) treatment. Downregulating glutathione (GSH) synthesis can decrease antioxidant protection with other antioxidative enzyme inhibitors including peroxidases (PRx), superoxide dismutase (SOD), and glutathione peroxidase (GPx).

Elevated levels of ROS are common hallmarks of cancer progression and resistance to treatment [41,42]. High ROS burden in SDHB deficient solid tumors offers a promising target for treatment. Although there are several pathways that activate ROS production, at the present time, there is little evidence to prove its effectiveness as an antiproliferative approach in *SDHB*-silenced PHEOs/PGLs in vitro and in animal models [34,35,37]. The benefits of high-dose ascorbic acid treatment in *SDHB^KD^* models resulting in the Fenton reaction (due to iron accumulation and consequent ROS production [34]) is a potential way to disrupt redox imbalance. For example, doxorubicin can induce iron-mediated increases in ROS [43], and this drug was previously found to limit in vitro growth of MTT cells that are considered to be a more aggressive model [44]. Fliedner et al. suggested that this may be linked to increased ROS production if compared to MPC cells [45]. However, the authors focused on the hypoxia signaling pathway rather than ROS production in the doxorubicin study [46], and thus the effectiveness of the drug in *SDHx*-models has yet to be verified. Other iron-dependent ROS generators such as dihydroartemisinin, erastin, and sulfasalazine [47], and/or the combination of these drugs with a high-dose ascorbic acid treatment, could potentially serve as a future iron/ROS-targeted strategy for *SDHx*-mutated tumors (Figure 3).

Tyrosine kinase inhibitors (TKIs) have been extensively studied for their antitumoral properties. TKIs target functions of different cellular compartments and signaling pathways. Certain TKIs induce mitochondrial dysfunction (i.e., uncoupling components of the electron transport chain), resulting in a drop in mitochondrial membrane potential and increased ROS production [48,49,50,51,52]. Treatment with certain TKIs such as imatinib or erlotinib was not effective in PHEO/PGL [53]. However, the insignificant findings of this study may be partially explained by the limited number of PHEO/PGL cases and lack of cases with *SDHB* mutations associated with ROS-mediated anticancer effects as well as by the specificity of certain TKIs. Indeed, treatment with a different inhibitor, sunitinib, led to a reduction in tumor size, stabilization of disease, and improvement in hypertension among *SDHB*-mutated PHEO/PGL patients [54], warranting further evaluation of this compound. While mitochondrial complexes I and III are relevant producers of ROS themselves [3], targeted disruption of oxidative phosphorylation was shown to increase ROS levels in several cancer models [55,56,57,58,59,60,61]. Targeting CI with metformin in rat PHEO cells (PC12 cells) revealed the antiproliferative potential of this drug [62,63] in vitro, however, these results have yet to be confirmed in vivo. Similarly, Florio et al. showed that metformin treatment promoted oxidative metabolism and decreased proliferation in cells isolated from *SDHC-* and *SDHD-*mutated patients [37]. Furthermore, rotenone, an inhibitor of CI, induced rat PHEO cell (PC12 cells) apoptosis by ROS production [64]. Even though these drugs have not been extensively tested in *SDHx*-mutated models, further accumulation of ROS could lead to ROS overload in mutated cells, resulting in cell apoptosis. Other CI and CIII inhibitors have yet to be tested (Figure 3).

NRF2 was found to be a promising therapeutic target in other neoplasms [65]. Targeting NRF2-dependent GSH synthesis was effective in the metastatic model of *SDHB*-silenced PHEO/PGL [35], thus, more strategies to prevent the overall production and/or availability of GSH in tumor cells may be beneficial in future PHEO/PGL therapies. Buthionine sulfoximine, an inhibitor of the enzyme glutamate cysteine ligase (required for GSH synthesis), depletes GSH, and exhibits anticancer activity [6]. When considering the benefits of GSH synthesis disruption in *SDHB^KD^* cells, we hypothesize that targeting other antioxidant pathways could be a potential strategy to expose cells to endogenously produced ROS. For example, it has been shown that MTT cells, more aggressive mouse PHEO cells derived from MPC cells, produce higher amounts of ROS when compared to MPC cells. This is accompanied by an increase in superoxide dismutase 1 [45] and such an increase in antioxidant protection can thus be hypothesized as a consequence of ROS accumulation in *SDHx*-mutated PHEO/PGL models. Similarly, *SDHB-*mutated PHEO/PGL showed increased expression of superoxide dismutase 2 when compared to *VHL*-mutated PHEO/PGL [45], which may play a role in the malignancy rates of patients with these mutations [66]. Thus, enzyme inhibitors may enable overproduction of ROS with consequent tumor cell death (Figure 3).

## 5. Conclusions

In conclusion, patients with *SDHB*-mutated PHEO/PGL have a higher likelihood of metastatic disease with limited therapeutic options and poor prognosis. Data regarding anticancer ROS-related drugs as potential therapeutic candidates for *SDHB*-mutated PHEO/PGL are still very limited. More studies are needed to evaluate the effectiveness and safety of these drugs in PHEO/PGL patients. Given the accumulation of promising evidence, ROS targeting may become an effective anticancer therapy in PHEO/PGL as well as other tumor types in the near future.
